# Cannabinoid WIN55,212-2 reprograms monocytes and macrophages to inhibit LPS-induced inflammation

**DOI:** 10.3389/fimmu.2023.1147520

**Published:** 2023-03-16

**Authors:** Mario Pérez-Diego, Alba Angelina, Leticia Martín-Cruz, Andrés de la Rocha-Muñoz, Angel Maldonado, Carmen Sevilla-Ortega, Oscar Palomares

**Affiliations:** ^1^ Department of Biochemistry and Molecular Biology, School of Chemistry, Complutense University of Madrid, Madrid, Spain; ^2^ Autonomous University of Madrid, Madrid, Spain

**Keywords:** cannabinoids, WIN55,212-2, monocyte differentiation, macrophage polarization, immunomodulation, anti-inflammatory, metabolic and epigenetic reprogramming

## Abstract

**Introduction:**

Chronic or uncontrolled activation of myeloid cells including monocytes, macrophages and dendritic cells (DCs) is a hallmark of immune-mediated inflammatory disorders. There is an urgent need for the development of novel drugs with the capacity to impair innate immune cell overactivation under inflammatory conditions. Compelling evidence pointed out cannabinoids as potential therapeutic tools with anti-inflammatory and immunomodulatory capacity. WIN55,212-2, a non-selective synthetic cannabinoid agonist, displays protective effects in several inflammatory conditions by mechanisms partially depending on the generation of tolerogenic DCs able to induce functional regulatory T cells (Tregs). However, its immunomodulatory capacity on other myeloid cells such as monocytes and macrophages remains incompletely understood.

**Methods:**

Human monocyte-derived DCs (hmoDCs) were differentiated in the absence (conventional hmoDCs) or presence of WIN55,212-2 (WIN-hmoDCs). Cells were stimulated with LPS, cocultured with naive T lymphocytes and their cytokine production and ability to induce T cell responses were analysed by ELISA or flow cytometry. To evaluate the effect of WIN55,212-2 in macrophage polarization, human and murine macrophages were activated with LPS or LPS/IFNγ, in the presence or absence of the cannabinoid. Cytokine, costimulatory molecules and inflammasome markers were assayed. Metabolic and chromatin immunoprecipitation assays were also performed. Finally, the protective capacity of WIN55,212-2 was studied in vivo in BALB/c mice after intraperitoneal injection with LPS.

**Results:**

We show for the first time that the differentiation of hmoDCs in the presence of WIN55,212-2 generates tolerogenic WIN-hmoDCs that are less responsive to LPS stimulation and able to prime Tregs. WIN55,212-2 also impairs the pro-inflammatory polarization of human macrophages by inhibiting cytokine production, inflammasome activation and rescuing macrophages from pyroptotic cell death. Mechanistically, WIN55,212-2 induced a metabolic and epigenetic shift in macrophages by decreasing LPS-induced mTORC1 signaling, commitment to glycolysis and active histone marks in pro-inflammatory cytokine promoters. We confirmed these data in *ex vivo* LPS-stimulated peritoneal macrophages (PMΦs), which were also supported by the *in vivo* anti-inflammatory capacity of WIN55,212-2 in a LPS-induced sepsis mouse model.

**Conclusion:**

Overall, we shed light into the molecular mechanisms by which cannabinoids exert anti-inflammatory properties in myeloid cells, which might well contribute to the future rational design of novel therapeutic strategies for inflammatory disorders.

## Introduction

Inflammation is a vital defense mechanism that can be initiated by the immune system after detecting invading pathogens and/or cellular damage. Inflammatory responses are orchestrated to eliminate the cause of injury and to initiate healing upon tissue damage. Alterations of these tightly regulated processes are associated with different immune-mediated diseases and inflammatory disorders such as autoimmune and allergic diseases, autoinflammatory syndromes or sepsis (1, 2). Myeloid cells such as conventional dendritic cells (DCs), monocytes and macrophages are specialized in the initiation and amplification of inflammatory responses. These cells are equipped with a large battery of pattern-recognition receptors (PRRs) to sense a plethora of harmful pathogens and damage-associated stimuli, which allows their activation, initiation of inflammatory responses and spreading of the alarm throughout the body (1, 3, 4). Compelling experimental evidence shows that inflammatory responses orchestrated by myeloid cells are closely linked to their metabolic profile (5, 6). For example, the activation of mammalian target of rapamycin complex 1 (mTORC1) on myeloid cells promotes anabolic metabolism and shift from glycolysis towards lactic fermentation as a faster energetic source to induce and sustain the functional adaptations required under a pro-inflammatory scenario (6). Under certain pathological conditions, the chronic or uncontrolled activation of myeloid cells significantly contributes to perpetuate inflammation and tissue damage, which leads to the main clinical manifestations associated to such diseases (2, 7). Although current immunosuppressive therapies represent the mainstay of treatment for many inflammatory conditions, their clinical efficacy is still limited for many patients and their overuse is associated with significant adverse effects (8). Therefore, the discovery of novel molecules able to target myeloid cells and dampen inflammation as well as the better understanding of their mode of action at the molecular level might well contribute to the development of alternative therapeutic interventions for many immune-mediated and inflammatory diseases.

The endocannabinoid system (ECS) is a complex signaling network encompassing the cannabinoid receptors (CBRs), the endogenous cannabinoids (anandamide, AEA) and 2-arachidonoylglycerol, 2-AG) and the enzymes involved in their synthesis and degradation (9, 10). In addition, phytocannabinoids from *Cannabis sativa L.*, marijuana, (Δ^9^-tetrahydrocannabinol, THC) and synthetic cannabinoids (WIN55,212-2, HU210, or HU308) also activate CBRs regulating proliferation, differentiation, cell survival, metabolism or immunity (9, 10). The therapeutic exploitation of the ECS is an emerging field of research, and different pre-clinical and clinical studies point out cannabinoids as potential therapeutic tools for inflammatory diseases (9–12). At this regard, we have previously demonstrated that human DCs express functional CBRs and that the synthetic cannabinoid WIN55,212-2 induces anti-inflammatory immune responses by promoting metabolic reprogramming in fully differentiated DCs, which eventually led to the generation of functional regulatory T cells (Tregs) with suppressive capacity both *in vitro* and *in vivo* (13). We have also shown that WIN55,212-2 is able to restore rhinovirus-induced bronchial epithelial barrier disruption (14). However, the potential capacity of WIN55,212-2 to regulate the generation of human monocyte-derived DCs (hmoDCs) and its anti-inflammatory properties on other myeloid cells such as macrophages remains elusive. Herein, we show for the first time that the differentiation of hmoDCs in the presence of WIN55,212-2 generates tolerogenic DCs (WIN-hmoDCs) that are less responsive to lipopolysaccharide (LPS) stimulation and able to prime functional Tregs. We also uncover that WIN55,212-2 inhibits the pro-inflammatory M1 activation in THP-1 Macrophages (THP-1 MΦs) and primary cultures of human monocyte-derived macrophages by mechanisms depending on metabolic and epigenetic rewiring. WIN55,212-2 protects from LPS-induced sepsis *in vivo* and limits LPS-induced metabolic reprogramming of *ex vivo* stimulated peritoneal macrophages (PMΦs). Our data enhance the knowledge about the molecular mechanisms by which cannabinoids exert anti-inflammatory properties in myeloid cells, which might help to the development of novel therapeutic interventions for inflammatory diseases.

## Materials and methods

### Material, media and reagents

Cell cultures were growth in RPMI 1640 medium (Lonza) supplemented with 10% heat‐inactivated fetal bovine serum (FBS, Hyclone), 100 μg/mL normocin (*Invivo*Gen), 50 μg/mL penicillin‐streptomycin, 1% nonessential amino acids, 1% MEM vitamins and 1 mmol/L sodium pyruvate (all from Life Technologies).

*In vitro* cell activation was performed with LPS from *Escherichia coli* (O127:B8, Sigma-Aldrich) and/or IFNγ (PeproTech). In *ex vivo* and *in vivo* experiments, LPS from *E. coli* (O155:B5, Sigma-Aldrich) was employed. The cannabinoid agonist WIN55,212-2 (Sigma-Aldrich) was used. The specific doses and time points are specifically detailed for each corresponding experiment.

### Generation of human monocyte-derived dendritic cells and WIN-hmoDCs

Peripheral blood mononuclear cells (PBMCs) were isolated from buffy coats of healthy anonymous donors (Transfusion Centre of Madrid, under the approved code “PODIS09”) by Ficoll Paque density-gradient. Then, monocytes were purified by positive magnetic isolation using anti-human CD14 microbeads and AutoMACS technology (Miltenyi Biotec). To generate hmoDCs, monocytes were seeded at 1x10^6^ cells/mL concentration and differentiated in the presence of 100 ng/mL of granulocyte-macrophage colony-stimulating factor (GM-CSF) and 100 ng/mL IL-4 (Preprotech) for 6 days. To generate WIN-hmoDCs, WIN55,212-2 (Sigma-Aldrich) at a final concentration of 50 nM was added at days 0 and 4 of the hmoDCs differentiation.

### Coculture experiments

Peripheral blood naïve CD4^+^ T cells were purified from PBMCs obtained from buffy coats of healthy donors using the “Naïve CD4^+^ T Cell Isolation Kit” (Miltenyi Biotec). LPS-stimulated hmoDCs and WIN-hmoDCs were cocultured with allogeneic naïve CD4^+^ T cells (hmoDC:T cell ratio of 1:5) during 5 days. Then, cell-free supernatants were collected for cytokine quantification and cells were stained for flow cytometry analysis.

### Differentiation of THP-1 MΦs and primary human monocyte-derived macrophages

THP-1 and THP-1 XBlue cell lines (*Invivo*Gen) were seeded at 0,25x10^6^ cells/mL and stimulated with 10 ng/mL phorbol 12-myristate 13-acetate (PMA, Sigma-Aldrich) for 24 h. Then, culture media was removed and fresh media was added. After resting for 24 h, cells were stimulated with medium or 100 ng/mL LPS from *E. coli* (O127:B8, Sigma-Aldrich) + 50 ng/mL IFNγ (Preprotech) (LPS/IFNγ) during 24h to generate M0 and M1 polarized THP-1 MΦs, respectively. To generate primary human macrophages, monocytes were purified from PBMCs as previously described (15, 16), seeded at 0,5x10^6^ cells/mL and cultured in the presence of 100 ng/mL of GM-CSF (Preprotech) for 7 days. Cultures were supplemented with GM-CSF every second day.

### NF-κB/AP-1 activation assay

The evaluation of NF-κB/AP-1 activation in human macrophages was performed using the THP-1 XBlue™ monocytic cell line (*Invivo*Gen), which is stably integrated with a NF-κB/AP-1 inducible secreted embryonic alkaline phosphatase (SEAP) reporter construct. Briefly, THP-1 XBlue monocytes were differentiated into human THP-1 MΦs as described above. Then, after incubation with the indicated stimuli, the activation of NF-κB/AP-1 was determined in cell-free supernatants using a SEAP colorimetric detection reagent called QUANTIBlue™.

### Cytokine quantification

Concentrations of human TNFα, IL-1β, IL-6, IL-10, IL-5, IFNγ and murine IL-6 in cell-free supernatants were quantified using sandwich ELISA cytokine kits from BD Biosciences. Murine TNFα and IL-1β levels were assessed by sandwich ELISA using specific kits from Invitrogen. In all cases, the manufacturer protocols were followed with minor modifications.

### Flow cytometry

For flow cytometry determinations, monoclonal antibodies were purchased from BioLegend: human anti-FOXP3-Alexa Fluor 488 (A488), anti-CD127-phycoerythrin (PE), anti-CD25-allophycocyanin (APC), anti-CD4-peridinin chlorophyll protein complex (PerCP), anti-HLA-DR-Fluorescein-5-isothiocyanate (FITC) anti-CD86-PE and anti-CD83-APC. Staining for CB1 was performed using the HU210-A488 fluorescent probe as previously described (15), A488 alone was use as control. For the detection of CB2 receptor, a first incubation with the primary CB2 polyclonal antibody (PA1744, Invitrogen) was followed by the incubation with an A488-conjugated goat anti-rabbit secondary antibody (Invitrogen). Cell viability was analyzed by propidium iodide (PI, Life technologies) or eFluor660 (Invitrogen) staining. For surface staining, cells were harvested, washed in 2 mM PBS/EDTA with 0.5% bovine serum albumin (BSA) and stained in the darkness for 15 min at room temperature with the corresponding fluorescence-labelled or isotype control antibodies. For the analysis of FOXP3 expression in human T cells cocultured with hmoDCs, a first surface staining with anti-human CD4-PerCP, CD127-PE, and CD25-APC antibodies was followed by subsequent fixation and permeabilization. Then, cells were stained with anti-human FOXP3–A488 according to the manufacturer’s recommendations. The corresponding isotype controls were included in every staining step. Flow cytometry analysis was performed with a FACSCalibur cytometer (Becton Dickinson) in the cytometry and fluorescence microscopy unit at the Complutense University of Madrid.

### Western Blot

After the corresponding treatments, cells were washed and lysed with RIPA buffer (Thermo Fisher Scientific) supplemented with Protease/Phosphatase Inhibitor Cocktail (Cell Signaling) and 1 mM PMSF (Sigma-Aldrich) for 30 min at 4°C. Protein was quantified with Micro BCA Kit (Pierce) following the manufacturer’s protocols. 10 μg of total protein from cell lysates were separated in 10% SDS-polyacrylamide gel electrophoresis (SDS-PAGE) and transferred onto a nitrocellulose membrane (Bio-Rad Laboratories). The membrane was blocked with 5% BSA, 0.1% Tween-20 in Tris buffered saline for 1 h and incubated with the indicated primary antibodies: phospho-Akt (Ser473) (1:1000, Cell Signaling), phospho-p70 S6 Kinase (Thr389) (1:750, Cell Signaling), and β-actin (1:15000, Sigma-Aldrich). Then, the membrane was washed and incubated with goat anti-rabbit (1:4000; Bio-Rad Laboratories) or goat anti-mouse (1:2500, Pierce) conjugated with horseradish peroxidase (HRP) as a secondary antibody. The signal was developed with Clarity Western ECL Substrate, detected in a Chemidoc XRS System and analyzed with Image Lab Software (all from Bio-Rad). Briefly, for the quantification of the images obtained, same-size regions of interest (ROIs) were displayed over the different bands and the intensity/mm^2^ of each ROI was quantified. After removal of the background value, the quantification of each band was then relativized to the one of its corresponding loading control (β-actin).

### Metabolic studies

The determination of glucose concentration in cell-free culture supernatants was assessed by using the Glucose (GO) Assay Kit (Sigma-Aldrich). The metabolic rate was derived mathematically as the percentage of glucose consumed relative to its concentration in medium without cells (2 mg/mL). Lactate concentration was measured in perchloric acid deproteinized culture supernatants by means of a two-step coupled lactate oxidase (Sigma-Aldrich) and HRP (Fisher Scientific) enzymatic reactions. Briefly, lactate was oxidized producing H_2_O_2_ which was coupled to the conversion of Amplex Red reagent (Fisher Scientific) to fluorescent resorufin by HRP. The fluorescence intensity of resorufin was analyzed using FLUOstar OPTIMA fluorescence reader (BMG LabTech).

Real-time metabolic profiling of macrophages was performed using a Seahorse XF HS Mini Analyzer (Agilent) following conventional protocols for adherent cells. Briefly, human macrophages were harvested at day 5 of differentiation and seeded (50x10^3^ cells/well) in XFp Cell Culture Miniplates with cRPMI supplemented with 100 ng/mL GM-CSF. Cells were incubated 24 h and then activated for 18 h with LPS or LPS+WIN55,212-2 (LPS+WIN), 10 ng/mL and 10 μM respectively. For peritoneal macrophage experiments, mice peritoneal cells were seeded (400x10^3^ cells/well) in XFp Cell Culture Miniplates with cRPMI supplemented with 20 ng/mL M-CSF and incubated for 3h. Peritoneal macrophages were purified by adherence through three consecutive washing steps and stimulated for 18 h with LPS or LPS+WIN55,212-2 (LPS+WIN), 10 ng/mL and 10 μM respectively. After stimulation, cRPMI medium was removed, cells were washed and cultured with XF DMEM medium supplemented with 10 mM glucose, 1 mM pyruvate and 2 mM glutamine in a CO_2_-free incubator for at least 1 h at 37°C before the assay. To accurately monitor glycolysis, we analysed the glycolytic proton efflux rate (glycoPER) at three consecutive stages: basal conditions (no drugs administrated), inhibition of the electron transport chain (0,5 µM Rotenone, ROT) and (0,5 µM Antimycin A, AA) and inhibition of glycolysis (50 mM 2-deoxy-D-glucose, 2-DG) (Sigma-Aldrich).

### Quantitative PCR

The RNA from collected samples was isolated with RNeasy Mini Kit (Qiagen) according to the manufacturer’s protocol. Next, complementary DNA was generated with a PrimeScript RT Reagent Kit (Takara Bio). Finally, quantitative PCR (qPCR) was performed by using FastStart Universal SYBR Green Master (Roche). The sequences of the primer pairs used are listed in **Supplementary Table 1**.

### Chromatin Immunoprecipitation

For chromatin immunoprecipitation (ChIP), cells were harvested and fixed after stimulation. Samples were lysed and sonicated using a Branson 1200 Ultrasonic Cleaner to obtain chromatin fragments of optimal size (300-800 bp). Sheared chromatin was incubated using 2 μL of anti-H3K27ac antibody (Abcam, ab4729) or 2 μL of anti-IgG antibody (Millipore, 12-370), and immunoprecipitated with Dynabeads A (Invitrogen, 10001D) and Dynabeads G (Invitrogen, 10003D) magnetic beads. ChIP samples were de-cross-linked with proteinase K (Thermo Fisher Scientific) at 65°C for 4 h. Then, DNA was purified with the DNA Clean Kit & Concentrator TM-5 (Zymo Research). Quantitative analysis of the purified ChIP and Input DNAs was performed by using FastStart Universal SYBR Green Master (Roche) by real time qPCR.

### *Ex vivo* culture of PMΦs

Female BALB/c mice were anesthetized using isoflurane and subjected to intraperitoneal lavage with 3 mL of 20 mM PBS/EDTA. The peritoneal cells were then washed, seeded in medium at 0,5x10^6^ cells/mL and allowed to rest for 3h. Then, PMΦs were isolated by adherence after three consecutive washing steps and treated with the indicated stimuli.

### *In vivo* LPS-induced sepsis model

All mice procedures included in this study were reviewed and ethically approved by Universidad Complutense de Madrid (UCM) and Comunidad Autónoma de Madrid (CAM) within the context of project SAF-2017-84978-R and PID2020-114396RB-I00 (*CAM:ref.10/250312.9/18 and CAM:ref.10/465020.9/21*). 6-week-old female BALB/c mice (Charles River) were randomly assigned into 4 groups. Then, mice were intraperitoneally injected with vehicle (PBS+DMSO), WIN55,212-2 (5 mg/kg), LPS (20 mg/kg), or LPS plus WIN55,212-2, respectively. Blood samples were collected 12 h after LPS administration *via* retro-orbital bleeding using lime grass Pasteur pipettes and microtainer tubes (Beckton Dickinson). To collect serum, samples were centrifuged at 10,000 rpm for 10 min at room temperature. Then, the presence of bilirubin in mice serum was determined by its absorbance at 450 nm, lactate dehydrogenase (LDH) activity was analyzed using CyQUANT LDH Cytotoxicity Assay Kit (Fisher) and the concentration of TNFα, IL-1β and IL-6 was measured by ELISA.

### Statistical analysis

Statistical analyses were performed with GraphPad Prism software (version 6.0) by using Paired t test, Unpaired t test, Wilcoxon test, One-way ANOVA or Spearman test. Significance was defined as: * P < 0.05, ** P < 0.01, *** P < 0.001 and **** P < 0.0001.

## Results

### Monocyte-derived dendritic cells differentiated in the presence of WIN55,212-2 display anti-inflammatory and tolerogenic features

**Figure 1:**
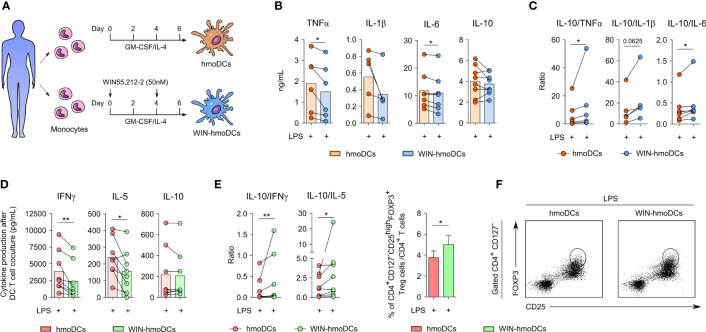
WIN-hmoDCs display anti-inflammatory and tolerogenic properties upon LPS stimulation. **(A)** Differentiation protocol of hmoDCs and WIN-hmoDCs from monocytes. **(B, C)** cytokine production **(B)** and ratios **(C)** of conventional hmoDCs and WIN-hmoDCs after LPS stimulation (100 ng/mL) (n=5-8). **(D, E)** cytokines **(D)** and ratios **(E)** produced by allogeneic naive CD4^+^ T cells primed by LPS-stimulated hmoDCs or WIN-hmoDCs after 5 days of coculture (n=8). **(F)** Percentage (left), and representative dot plots (right) of induced CD4^+^CD25^high^CD127^-^FOXP3^high^ Tregs by allogeneic LPS-stimulated hmoDCs or WIN-hmoDCs (n=7). Values are shown as mean ± SEM. Statistical significance was determined by Paired t test **(B, D, F)** or Wilcoxon test **(C, E)**. *P<0.05, **P<0.01.

To determine the capacity of WIN55,212-2 to modulate the phenotype and function of human monocyte-derived DCs (hmoDCs), monocytes were differentiated into DCs under conventional protocols in the absence or presence of this synthetic cannabinoid (**Figure 1A**). First, the expression of CBRs in monocytes was confirmed at both mRNA and protein level (**Supplementary Figure 1**). Then, we used 50 nM of WIN55,212-2 during the differentiation process to generate WIN-hmoDCs, as this concentration showed the most effective results without affecting cell viability during dose-response optimization experiments (**Supplementary Figure 2**). After 18 h of LPS stimulation, conventional hmoDCs produced higher amounts of the pro-inflammatory cytokines TNFα, IL-1β and IL-6 than WIN-hmoDCs (**Figure 1B**). No significant differences were observed in the production of the anti-inflammatory cytokine IL-10. The IL-10/TNFα, IL-10/IL-1β and IL-10/IL-6 cytokine ratios were significantly higher in LPS-stimulated WIN-hmoDCs than conventional hmoDCs (**Figure 1C**), indicating that WIN55,212-2 reprograms the differentiation of monocytes towards DCs displaying anti-inflammatory features upon LPS stimulation. Interestingly, no differences in the expression of HLA-DR or the costimulatory molecules CD83 or CD86 were observed between conventional hmoDCs and WIN-hmoDCs (**Supplementary Figure 3**), suggesting that WIN-hmoDCs might well preserve their capacity to antigen presentation and T cell activation. To further assay the capacity of the generated WIN-hmoDCs to polarize T cell responses upon LPS stimulation, we performed allogeneic coculture experiments with naïve CD4^+^ T cells. LPS-stimulated WIN-hmoDCs generated T cells that produced significantly lower amounts of IFNγ and IL-5 than LPS-activated hmoDCs, without significant differences observed in the production of IL-10 (**Figure 1D**). The IL-10/IL-5 and IL-10/IFNγ ratios were significantly higher in T cells generated by WIN-hmoDCs than conventional hmoDCs (**Figure 1E**), suggesting the generation of Tregs by WIN-hmoDCs. Supporting these data, the frequency of CD4^+^ CD127^-^ CD25^high^ FOXP3^high^ Tregs generated by LPS-activated WIN-hmoDCs was significantly higher than by LPS-stimulated conventional hmoDCs (**Figure 1F**). Collectively, these data indicate that the presence of WIN55,212-2 during monocyte differentiation promotes the generation of DCs with anti-inflammatory properties upon LPS stimulation and with increased capacity to polarize Tregs.

### WIN55,212-2 inhibits the activation of pro-inflammatory M1 human macrophages

**Figure 2:**
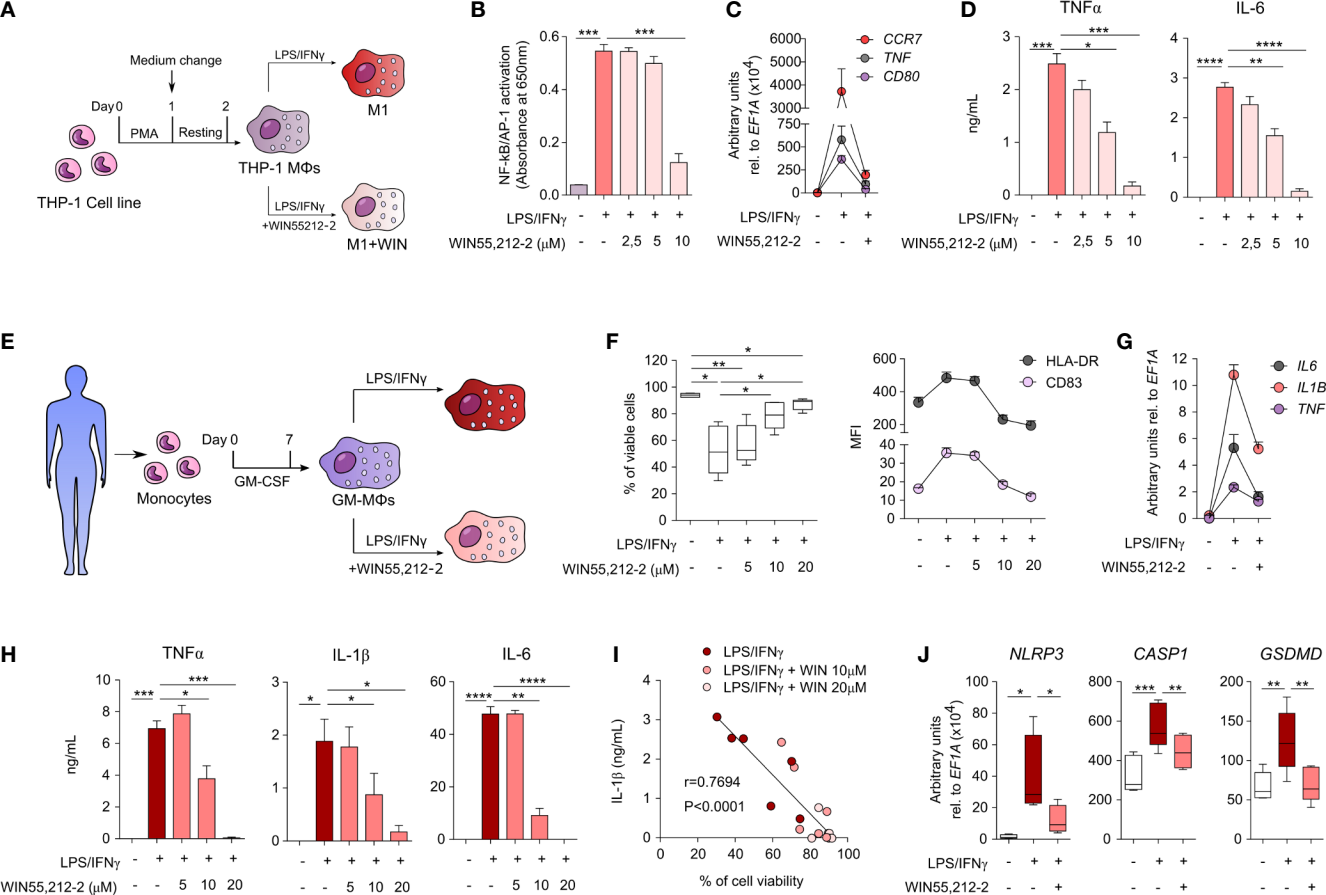
WIN55,212-2 inhibits pro-inflammatory activation of human macrophages. **(A)** THP-1 MΦs generation and activation protocol. **(B)** NF-κB/AP-1 activation in THP-1 XBlue MΦs after 18h of stimulation with medium, LPS/IFNγ (100 ng/mL and 50 ng/mL, respectively) or LPS/IFNγ plus the indicated doses of WIN55,212-2 (n=6). **(C)** mRNA expression levels of the indicated genes after 18h of stimulation of THP-1 MΦs with medium, LPS/IFNγ or LPS/IFNγ plus WIN55,212-2 (10 μM) (n=5). **(D)** Cytokine production by THP-1 MΦs stimulated with medium, LPS/IFNγ or LPS/IFNγ plus the indicated doses of WIN55,212-2 (n=6). **(E)** Protocol of human GM-MΦs differentiation and activation. **(F)** Percentage of viable cells (left) and mean fluorescence intensity (MFI) of the indicated markers (right) after GM-MΦs stimulation with medium, LPS/IFNγ (100 ng/mL and 50 ng/mL) or LPS/IFNγ plus the indicated doses of WIN55,212-2 (n=6). **(G)** mRNA expression levels of the indicated cytokines after 4h stimulation of GM-MΦs with medium, LPS/IFNγ or LPS/IFNγ plus WIN55,212-2 (20 μM) (n=5). **(H)** Cytokine production after 18h stimulation of GM-MΦs with medium, LPS/IFNγ or LPS/IFNγ plus the indicated doses of WIN55,212-2 (n=6). **(I)** Correlation between IL-1β production and GM-MØ viability after stimulation with LPS/IFNγ and LPS/IFNγ plus the indicated doses of WIN55,212-2 (n=6). **(J)** mRNA expression levels of the indicated genes after 4h stimulation of GM-MΦs with medium, LPS/IFNγ or LPS/IFNγ plus WIN55,212-2 (20 μM) (n=5). Values are shown as mean ± SEM. Statistical significance was determined by One-way ANOVA **(B, D, F, H, J)** or Spearman test **(I)**. *P<0.05, **P<0.01, ***P<0.001 and ****P<0.0001.

To investigate the effects of WIN55,212-2 in human macrophages under inflammatory conditions, we first generated human macrophages from the THP-1 monocytic cell line (THP-1 MΦs) and stimulated them with medium (control), LPS/IFNγ alone (M1) or in the presence of WIN55,212-2 (M1+WIN) (**Figure 2A**). WIN55,212-2 inhibited LPS/IFNγ-induced NF-κB/AP-1 activation in THP-1 MΦs in a dose dependent manner (**Figure 2B**). Supporting these data, WIN55,212-2 impaired the mRNA expression of the classical M1 markers *CCR7*, *TNF* or *CD80* induced by LPS/IFNγ stimulation in THP-1 MΦs (**Figure 2C**) and inhibited the production of the pro-inflammatory cytokines TNFα and IL-6 in a dose-dependent manner (**Figure 2D**).

To further verify these results, we generated primary human monocyte-derived macrophages as shown in **Figure 2E**, which we termed as GM-MΦs according to Murray et al. (17). The expression levels of CBRs in GM-MΦs was confirmed at both mRNA and protein level (**Supplementary Figure 4**). Next, GM-MΦs were stimulated with medium (control), LPS/IFNγ alone or in the presence of WIN55,212-2 at different doses during 18 h to monitor cell viability and activation status. The viability of GM-MΦs stimulated with LPS/IFNγ alone was significantly compromised compared to unstimulated cells. Interestingly, WIN55,212-2 dose-dependently enhanced cell viability of LPS/IFNγ-stimulated GM-MΦs (**Figure 2F**), suggesting the inhibition of LPS/IFNγ-induced inflammatory cell death pathways in human GM-MΦs. The cannabinoid also downregulated the LPS/IFNγ-induced expression of HLA-DR and the costimulatory molecule CD83 in a dose dependent manner (**Figure 2F**). Likewise, LPS/IFNγ-activated GM-MΦs treated with WIN55,212-2 reduced the production of the pro-inflammatory cytokines TNFα, IL-1β and IL-6 both at the mRNA (**Figure 2G**) and protein level (**Figure 2H**), thus confirming the potent anti-inflammatory profile. In all cases, LPS/IFNγ-induced IL-10 levels were also inhibited by WIN55,212-2 in a dose-dependent manner (**Supplementary Figure 5**), suggesting that IL-10-producing M2 phenotypes in human macrophages are not generated under the assayed conditions. Remarkably, we uncovered a significant inverse correlation between IL-1β production and cell viability in GM-MΦs stimulated under the different assayed conditions (**Figure 2I**), which prompted us to investigate whether WIN55,212-2 could interfere with inflammasome activation and pyroptotic-mediated cell death. For that, we quantified the mRNA expression levels of the inflammasome components *NLRP3* and *CASP1*, and the pore-forming protein *GSDMD* after macrophage activation under different conditions. LPS/IFNγ stimulation significantly enhanced the mRNA expression levels of all the assayed molecules, which were in all the cases significantly impaired by WIN55,212-2 (**Figure 2J**), confirming its potential capacity to rescue human macrophages from this inflammatory cell death pathway.

### WIN55,212-2 impairs the LPS-induced metabolic and epigenetic reprogramming in human macrophages

**Figure 3:**
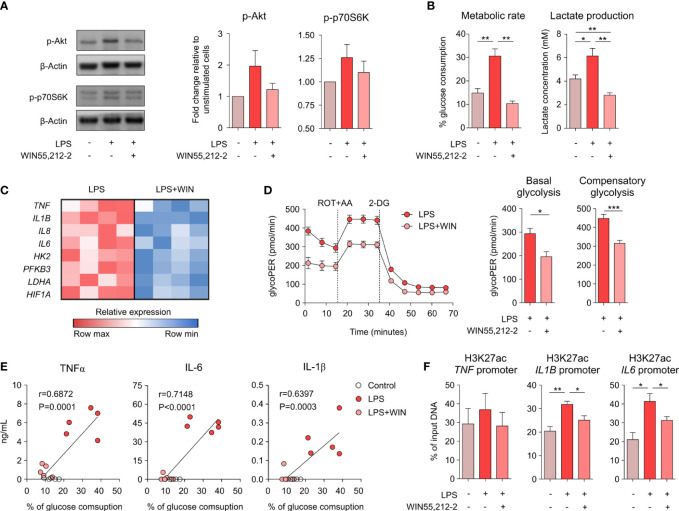
WIN55,212-2 impairs the metabolic and epigenetic reprogramming induced by LPS in human macrophages. **(A)** Representative western blot (left) and densitometric quantification (right) of GM-MΦs stimulated with medium, LPS (10 ng/mL) or LPS plus WIN55,212-2 (10 µM). The graph shows combined results from 30 and 60 min of stimulation (n=6 independent experiments with 4 different donors). **(B)** Percentage of glucose consumption and lactate concentration measured from cell-free supernatants of GM-MΦs stimulated with medium, LPS (10 ng/mL) or LPS plus WIN55,212-2 (10μM) (n=6). **(C)** Heat map showing the expression of pro-inflammatory cytokine and glycolysis-related genes in GM-MΦs stimulated with LPS or LPS+WIN55,212-2 during 4h (n=4). **(D)** Real-time analysis of glycolytic Proton Efflux Rate (glycoPER) of GM-MΦs after 18h stimulation with LPS or LPS plus WIN55,212-2 (LPS+WIN) at basal conditions and after sequential addition of rotenone plus antimycin A (ROT+AA) and 2-deoxyglucose (2-DG). Graphs representing basal and compensatory glycolysis are displayed (right) (n=6 of two independent experiments). **(E)** Correlation between TNFα, IL-1β or IL-6 with the % of glucose consumption of GM-MΦs stimulated with medium (Control), LPS or LPS+WIN (n=5). **(F)** Analysis of H3K27ac histone modifications at the promoter sites of the indicated genes in GM-MΦs stimulated with medium, LPS or LPS plus WIN55,212-2 for 4h (n=5). Values are shown as mean ± SEM. Statistical significance was determined by One-way ANOVA **(B, F)** Unpaired t test **(D)** or Spearman test **(E)**. *P<0.05, **P<0.01 and ***P<0.001.

To shed light into the molecular mechanisms underlying the anti-inflammatory capacity of WIN55,212-2 in human macrophages, we sought to investigate the potential effects of this cannabinoid on the metabolic pathways linked to the acquisition of inflammatory phenotypes in innate immune cells. For this set of experiments, we chose a lower dose of LPS for macrophage stimulation to avoid bias due to overactivation-induced cell death. Stimulation of GM-MΦs with LPS (10 ng/mL) mimics the previous LPS/IFNγ activation by significantly inducing the production of pro-inflammatory cytokines without affecting cell viability (**Supplementary Figure 6**). We initially analysed the mTORC1 signalling pathway as the key regulator of anabolic metabolism linked to inflammatory features in macrophages. WIN55,212-2 inhibited the LPS-induced phosphorylation of Akt and p70S6K (**Figure 3A**), indicating a reduced activation of the mTORC1 pathway and suggesting the potential regulation of LPS-induced metabolic reprogramming. The administration of WIN55,212-2 alone showed minimal changes in glucose uptake and lactate production (**Supplementary Figure 7**). Nonetheless, WIN55212,2 significantly reduced the high glucose consumption and the increased lactate production observed in LPS-stimulated GM-MΦs (**Figure 3B**), demonstrating the capacity of this cannabinoid to interfere with the glycolytic metabolism. Supporting these data, WIN55,212-2 significantly reduced the mRNA expression levels of the glycolysis-related genes *HK2*, *PFKB3*, *LDHA1* and *HIF1A* in LPS-stimulated macrophages, which was accompanied by significantly lower mRNA levels of the pro-inflammatory cytokines *TNF*, *IL1B*, *IL8* and *IL6* (**Figure 3C**). To further confirm these results, we performed functional metabolic experiments using a Seahorse bioanalyzer to monitor real-time glycolytic proton efflux rate (glycoPER) in LPS- or LPS + WIN55,212-2-stimulated GM-MΦs. Our data showed that commitment to glycolysis was significantly higher in LPS-stimulated than LPS+WIN55,212-2-stimulated GM-MΦs both at basal conditions (basal glycolysis) and after the inhibition of the electron transport chain (compensatory glycolysis) (**Figure 3D**). Remarkably, the production of TNFα, IL-1β and IL-6 by GM-MΦs stimulated with LPS or LPS+WIN55,212-2, directly correlated with the amount of glucose consumed by those cells (**Figure 3E**), indicating that the inhibition of the glucose uptake by human macrophages might well be connected to WIN55,212-2 anti-inflammatory effects. As recent evidence links LPS-induced glycolysis with epigenetic changes that govern pro-inflammatory cytokine production in macrophages (18, 19), we studied chromatin status near the *TNF*, *IL1B* and *IL6* gene promoters by quantifying the activation histone mark H3K27ac by ChIP analysis. LPS significantly enhanced the acetylation of H3K27 within the pro-inflammatory *IL1B* and *IL6* genes, which was significantly reduced by WIN55,212-2 (**Figure 3F**). A similar non-significant tendency was also observed within the *TNF* gene promoter. Collectively, these findings indicate that WIN55,212-2 inhibits the metabolic and epigenetic inflammatory reprogramming induced by LPS in human macrophages.

### WIN55,212-2 protects from LPS-induced sepsis *in vivo* and limits LPS-induced metabolic reprogramming of *ex vivo* stimulated peritoneal macrophages

**Figure 4:**
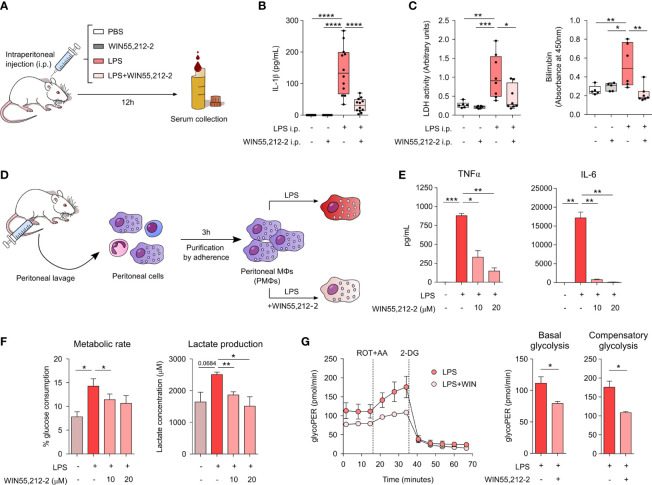
*In vivo* and *ex vivo* protective and anti-inflammatory potential of WIN55,212-2. **(A)** Schematic representation of the LPS-induced sepsis model. **(B)** Serum IL-1β levels after 12h of intraperitoneal (i.p.) administration of PBS (Control), WIN55,212-2 (5mg/kg), LPS (20mg/kg) or LPS plus WIN55,212-2 (n=7-12). **(C)** Assessment of the clinical parameters LDH and bilirubin in the serum of mice treated with the indicated conditions (n=5-8). **(D)** Protocol for peritoneal macrophages (PMΦs) isolation and activation. **(E)** Cytokine production by *ex vivo* stimulated PMΦs with medium, LPS (10 ng/mL) or LPS plus the indicated doses of WIN55,212-2 (n=4). **(F)** Percentage of glucose consumption and lactate concentration measured from cell-free supernatants of PMΦs treated with medium, LPS or LPS plus the indicated doses of WIN55,212-2 (n=6). **(G)** Real-time analysis of glycolytic Proton Efflux Rate (glycoPER) of PMΦs after 18h stimulation with LPS or LPS plus WIN55,212-2 at basal conditions and after sequential addition of rotenone plus antimycin A (ROT+AA) and 2-deoxyglucose (2-DG). Graphs representing basal as well as compensatory glycolysis are included on the right (n=3). Statistical significance was determined by One-way ANOVA or Unpaired t test. *P<0.05, **P<0.01, ***P<0.001 and ****P<0.0001.

To assess the potential *in vivo* anti-inflammatory properties of WIN55,212-2, we employed a mice model of LPS-induced sepsis. Mice were intraperitoneally injected with PBS, WIN55,212-2 or a lethal dose of LPS alone or in the presence of WIN55,212-2 (**Figure 4A**). After 12h, we collected mice serum and quantified the levels of IL-1β, lactate dehydrogenase (LDH) activity and bilirubin levels as hallmarks of the inflammatory response/inflammasome activation, cell death or organ injury, respectively. Intraperitoneal administration of LPS significantly enhanced the levels of serum IL-1β, which was significantly impaired by WIN55,212-2 (**Figure 4B**). Remarkably, not only inflammasome-dependent IL-1β release but the levels of other inflammatory mediators such as TNFα and IL-6 were also reduced by the treatment with the cannabinoid (**Supplementary Figure 8**). Similarly, increased levels of serum LDH and bilirubin were found in LPS-treated mice while mice receiving LPS+WIN55,212-2 showed significantly lower levels of both parameters (**Figure 4C**). These data confirmed the *in vivo* anti-inflammatory properties of WIN55,212-2 in the context of LPS-induce sepsis. As macrophages depict one of the main immune cell populations of the peritoneal cavity (20), we next sought to investigate their potential contribution into such protective anti-inflammatory effects. For that, we isolated peritoneal macrophages (PMΦs) and stimulated them *ex vivo* with medium (control), LPS or LPS+WIN55,212-2 (**Figure 4D**). WIN55,212-2 inhibited the production of TNFα and IL-6 by LPS-stimulated PMΦs in a dose-dependent manner (**Figure 4E**). Likewise, LPS-induced glucose consumption and lactate production was significantly impaired by WIN55,212-2, suggesting that this synthetic cannabinoid also inhibits LPS-induced metabolic rewiring in *ex vivo* stimulated PMΦs (**Figure 4F**). Supporting these data, real-time metabolic experiments showed a significantly reduced basal and compensatory glycolysis in LPS+WIN-stimulated PMΦs compared LPS-stimulated PMΦs, thus confirming our findings (**Figure 4G**). Collectively, these data show the *in vivo* anti-inflammatory capacity of WIN55,212-2 and its potential to immunomodulate tissue-specific macrophage populations during LPS-induced inflammatory conditions.

## Discussion

In this study, we uncover unpreceded mechanisms about how the synthetic cannabinoid WIN55,212-2 displays immunomodulatory and anti-inflammatory properties by regulating the differentiation of human monocytes into DCs and the activation of pro-inflammatory macrophages. We showed that tolerogenic WIN-hmoDCs are less responsive to LPS and prime functional Tregs. In addition, WIN55,212-2 impaired the pro-inflammatory polarization of human macrophages by inhibiting cytokine production, inflammasome activation and rescuing macrophages from pyroptotic cell death. Mechanistically, WIN55,212-2 induced a metabolic and epigenetic shift in macrophages by decreasing LPS-induced mTORC1 signaling, commitment to glycolysis and active histone marks in pro-inflammatory cytokine promoters. These data were confirmed in *ex vivo* stimulated PMΦs and supported by the anti-inflammatory capacity of WIN55,212-2 in a LPS-induced sepsis mouse model. Overall, we report novel insights into the anti-inflammatory capacity of WIN55,212-2 in myeloid cells, enhancing the knowledge on cell targets and mechanisms driving its potential beneficial effects in inflammatory conditions. Our findings might well contribute to open new avenues for the rational design of alternative cannabinoid-based therapeutic agents that are not able to cross the blood-brain barrier but retain anti-inflammatory/immunomodulatory properties on peripheral immune cells.

Monocytes are myeloid cells whose immune versatility relies on their capacity to differentiate into DCs or macrophages (4, 21). The environmental milieu to what monocytes are exposed is linked to the immunogenicity of the resulting differentiated cells. For example, the presence of stimuli such as dexamethasone (Dex), vitamin D3 (VitD3), rapamycin, or allergoid-mannan conjugates promotes the differentiation of monocytes into tolerogenic DCs or immunosuppressive macrophages (22–26). Conversely, monocytes exposed with whole heat-inactivated *Candida albicans* or β-glucans from its cell wall give raise to trained monocyte-derived macrophages (27–29). Here, we show that LPS-stimulated WIN-hmoDCs displayed reduced production of the pro-inflammatory cytokines TNFα, IL-1β and IL-6 without changes in IL-10. Supporting these data, previous studies demonstrated that hmoDCs generated in the presence of THC produced less IL-12 after activation. THC also reduced the phagocytic activity, maturation state and the capacity of the differentiated hmoDCs to activate T cells (30). Likewise, WIN-hmoDCs primed FOXP3^+^ Tregs and displayed reduced capacity to polarize both Th1 and Th2 responses. The induction and/or expansion of Tregs is essential to keep homeostasis in the context of immune-mediated and inflammatory diseases (31–33), which might be regulated by exogenous signals including cannabinoids like cannabidiol (CBD), JTE907 or O-1966 (34–36). We previously showed that fully differentiated conventional hmoDCs and blood DCs subsets stimulated *in vitro* with toll-like receptor ligands in the presence of WIN55,212-2 promote functional Tregs (13, 37, 38), which was further corroborated *in vivo* in LPS-induced sepsis, peanut-allergic sensitization and anaphylaxis models (13, 37). Our current data enhance the knowledge on how WIN55,212-2 could promote Tregs not only by directly acting on fully differentiated DCs, but also by imprinting tolerogenicity during monocyte differentiation into DC.

Macrophages are major producers of cytokines and chemokines that are essential for innate immune responses (3, 4). In this work, we focused on the capacity of WIN55,212-2 to impair pro-inflammatory macrophage polarization. Previous studies reported that AEA, 2-AG, THC or CBD inhibit the production of inflammatory mediators in macrophages (39–41). WIN55,212-2 impaired NF-κB activation and cytokine release in LPS-stimulated TLR3/TLR4-transfected HEK293 cells, hmoDCs and murine macrophages activated with oxidized-LDL (13, 42, 43). Consistent with these reports, we showed that WIN55,212-2 impaired NF-κB/AP-1-activation, decreased the expression of activation markers and the production of inflammatory cytokines in classically M1 activated THP-1 MΦs, which was comprehensively validated in primary human macrophages (GM-MΦs) and murine tissue-resident macrophages (PMΦs). WIN55,212-2 also restored GM-MΦs viability after LPS/IFNγ-induced inflammatory cell death, which correlated with a reduced release of IL-1β and impaired expression of *NLRP3*, *CASP1* and *GSDMD*. These findings suggest that WIN55,212-2 interferes with inflammasome activation and protects from pyroptotic macrophage death, which is congruent with the previous reported capacity of CBD or THC to prevent inflammasome-mediated signalling (41, 44). Nonetheless, we cannot rule out the potential contribution of WIN55,212-2 to interfere with other cell death pathways, since the recently coined term PANoptosis recognizes the complexity of programmed cell death signalling and the potential interplay between its molecular executors (45, 46). In fact, it was recently described that caspase 8 and other apoptotic proteins were involved in the IFNγ priming of macrophage TLR-induced death (47). The combination of IFNγ with pro-inflammatory cytokines, mainly TNFα, also induces macrophage death and perpetuates inflammation during cytokine shock syndrome (48). Herein, the cannabinoid WIN55,212-2 dose-dependently reduced the production of TNFα in LPS/IFNγ-treated GM-MΦs, suggesting its potential to prevent both cytokine storm and inflammatory cell death.

The anti-inflammatory capacity of cannabinoids has been linked with their ability to interfere with TLR-signalling and to induce metabolic reprogramming in immune cells (9, 49, 50). Our previous findings in human DCs indicate that the WIN55,212-2-mediated activation of CB1 as well as of the non-canonical cannabinoid receptor, peroxisome proliferator-activated receptor α (PPARα), leads to a tolerogenic profile characterized by AMPK-mediated induction of autophagy and promotion of oxidative phosphorylation over glycolysis (13). Herein, we show that WIN55,212-2 impaired LPS-induced phosphorylation of mTORc1’s upstream and downstream signalling molecules, Akt and p70S6K, in human macrophages. Aerobic glycolysis is the main supply for the energetic demands of pro-inflammatory activated macrophages after TLR-engagement (51, 52). Our real time metabolic experiments in LPS or LPS+WIN55,212-2-stimulated GM-MΦs showed reduced basal and compensatory glycolysis in the latter ones, indicating inhibition of TLR-induced metabolic rewiring. In this context, Lauterbach and others demonstrated that glucose uptake and glycolysis provide metabolic substrates that support LPS-induced inflammatory gene expression through epigenetic histone modifications (18, 19). LPS augments H3K27ac at *IL1B* and *IL6* gene promoters in GM-MΦs, which was impaired by WIN55,212-2, supporting that WIN55,212-2 may alter the LPS-induced metabolic-epigenetic axis in human macrophages. Beyond the potentiation of inflammatory cytokine expression, the induction of aerobic glycolysis also sustains inflammasome induction and its inhibition compromises IL-1β production (53–55). In addition, activation of mTORc1 pathway is linked to inflammasome activation, gasdermin D oligomerization and pyroptosis (56, 57). Altogether these data might well support our findings on the capacity of WIN55,212-2 to prevent inflammatory cell death.

Systemic inflammatory diseases with uncontrolled release of pro-inflammatory mediators (cytokine storm), are life-threatening conditions that remain poorly managed by current treatments (58–60). For instance, during endotoxin shock, the cooperative activation of inflammatory cytokine and cell death signalling drives severe tissue injury and multi-organ failure that may be eventually lethal (61, 62). Here, WIN55,212-2 abolished the LPS-induced increase of serum IL-1β in mice, confirming its previously reported *in vivo* anti-inflammatory capacity (13, 37, 42, 63–66). Inflammasome activation during sepsis aggravates organ injury and worsens prognosis. Suppression of inflammasome and pyroptosis protects mice from septic myocardial dysfunction, and clinical trial data shows reduced mortality after blockage of IL-1R in septic patients developing macrophage activation syndrome (MAS) (67, 68). Patients that develop MAS were characterized by hepatobiliary dysfunction and had significantly higher risk of death (69). We showed that WIN55,212-2-mediated decrease of serum IL-1β was associated with reduced levels of the myocardial and hepatic injury markers LDH and bilirubin. Remarkably, we showed that cytokine production of *ex vivo* stimulated PMΦs was dose-dependently modulated by WIN55,212-2 and associated with a markedly reduced glucose consumption and commitment to glycolysis. Our findings are congruent with the fact that inhibition of glycolysis with 2-DG mimicked WIN55,212-2 immunomodulatory effects in M1-activated murine alveolar macrophages and the capacity of WIN55,212-2 to protect mice from acute lung injury (63, 64).

Collectively, we uncover novel findings on the immunosuppressive capacity of WIN55,212-2 by acting on monocytes and macrophages and provide novel insights into the molecular mechanisms underlying such effects. Our *in vivo* data confirms WIN55,212-2 anti-inflammatory properties and highlights the important involvement of myeloid lineage cells in its potential therapeutic benefits. Our data might well contribute to pave the way for the development of alternative cannabinoid-based therapies for immune-mediated and inflammatory diseases.

## Data availability statement

The raw data supporting the conclusions of this article will be made available by the authors, without undue reservation.

## Ethics statement

All mice procedures included in this study were reviewed and ethically approved by Universidad Complutense de Madrid (UCM) and Comunidad Autoínoma de Madrid (CAM) within the context of project SAF-2017-84978-R and PID2020-114396RB-I00 (CAM:ref.10/250312.9/18 and CAM:ref.10/465020.9/21).

## Author contributions

Conceived and designed the study: OP. Performed the experiments: MP-D (human and mice experiments), AA (mice experiments and technical support for human experiments), LM-C, AR-M, AM, and CS-O (technical support for human experiments). Analyzed and discussed the data: MP-D, AA, LM-C, AR-M, AM, CS-O, and OP. Wrote the paper: MP-D and OP. All authors contributed to the article and approved the submitted version.

## Supplementary material

Supplementary Figure 1 Expression of CBRs in human monocytes. mRNA **(A)** and protein levels **(B)** of CB1 and CB2 in human monocytes was assayed by qPCR and flow cytometry (n=3). Values are shown as mean ± SEM.

Supplementary Figure 2 Viability **(A)** and relative cytokine production **(B)** of LPS-activated conventional hmoDCs or hmoDCs differentiated from monocytes in the presence of the indicated doses of WIN55,212-2 (n=2-5). Values are shown as mean ± SEM.

Supplementary Figure 3 Expression of costimulatory molecules in conventional hmoDCs and WIN-hmoDCs. Mean fluorescence intensity (MFI) of the indicated surface molecules expressed by LPS-activated conventional hmoDCs or WIN-hmoDCs (n=7). Values are shown as mean ± SEM.

Supplementary Figure 4 Expression of CBRs in human macrophages. mRNA **(A)** and protein levels **(B)** of CB1 and CB2 in human macrophages was assayed by qPCR and flow cytometry (n=3). Values are shown as mean ± SEM.

Supplementary Figure 5 WIN55,212-2 effect on IL-10 production. Graph shows IL-10 levels after stimulation of GM-MΦs with medium, LPS/IFNγ (100 ng/mL and 50 ng/mL) or LPS/IFNγ plus the indicated doses of WIN55,212-2 (n=6). Values are shown as mean ± SEM. Statistical significance was determined by One-way ANOVA. * P<0.05.

Supplementary Figure 6 Lower doses of LPS induces inflammatory activation of GM-MΦs without affecting cell viability. Percentage of viable cells **(A)** and cytokine production **(B)** after stimulation of GM-MΦs with medium, LPS (10 ng/mL) or LPS plus the indicated doses of WIN55,212-2 (n=6). Values are shown as mean ± SEM. Statistical significance was determined by One-way ANOVA. * P<0.05, ** P<0.01 and *** P<0.001.

Supplementary Figure 7 Metabolic changes induced by WIN55,212-2 administration in human macrophages. Percentage of glucose consumed **(A)** and lactate produced **(B)** by unstimulated or WIN55,212-2 activated GM-MOs (n=2). Values are shown as mean ± SEM.

Supplementary Figure 8 WIN55,212-2 inhibits cytokine production in LPS-induced septic mice. Serum levels of TNFα **(A)** and IL-6 **(B)** after 12h of intraperitoneal (i.p.) administration of PBS (Control), WIN55,212-2 (5mg/kg), LPS (20mg/kg) or LPS plus WIN55,212-2 (n=4-5). Values are shown as mean ± SEM. Statistical significance was determined by One-way ANOVA. * P<0.05, ** P<0.01, *** P<0.001 and **** P<0.0001.
