# Supplementary figures and images for: Cannabinoid WIN55,212-2 reprograms monocytes and macrophages to inhibit LPS-induced inflammation
Source: Front Immunol. 2023 Mar 16;14:1147520. doi: 10.3389/fimmu.2023.1147520 (PMC10060516; doi:10.3389/fimmu.2023.1147520)

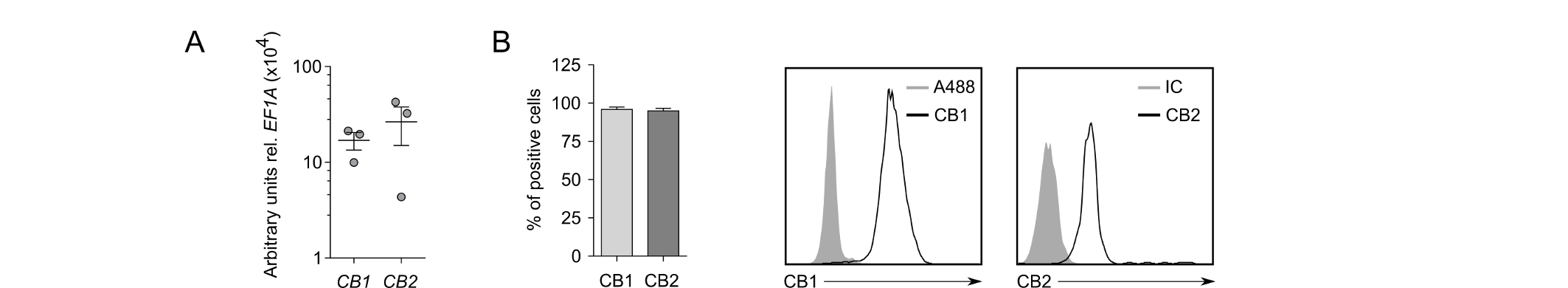

Supplement: Supplementary Figure 1 — Expression of CBRs in human monocytes. mRNA (A) and protein levels (B) of CB1 and CB2 in human monocytes was assayed by qPCR and flow cytometry (n=3). Values are shown as mean ± SEM. [file Image_1.tif]

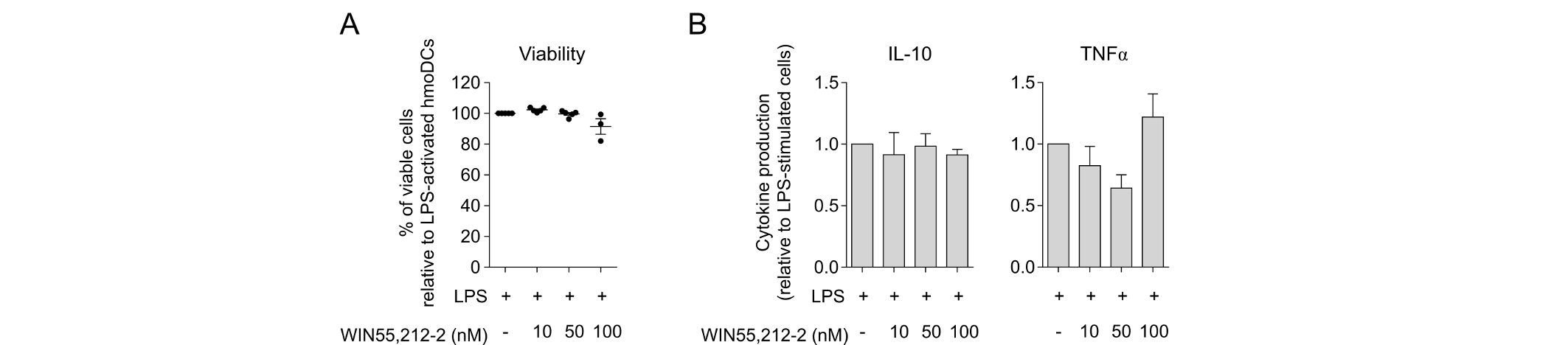

Supplement: Supplementary Figure 2 — Viability (A) and relative cytokine production (B) of LPS-activated conventional hmoDCs or hmoDCs differentiated from monocytes in the presence of the indicated doses of WIN55,212-2 (n=2-5). Values are shown as mean ± SEM. [file Image_2.tif]

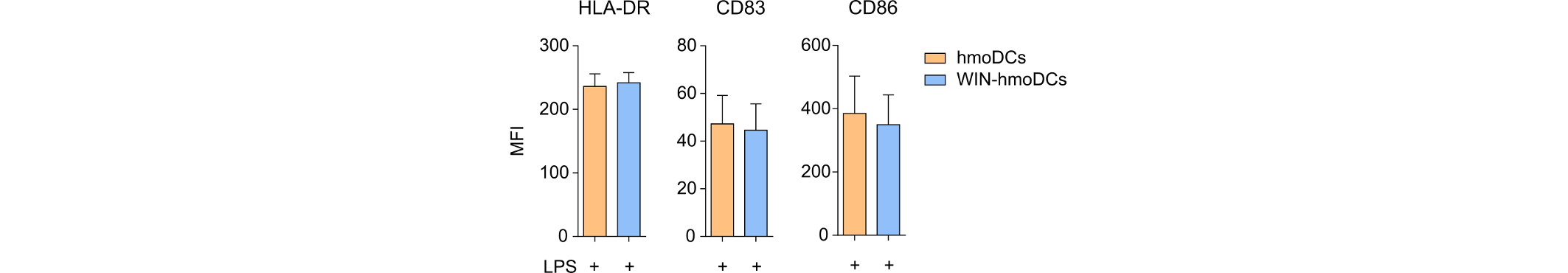

Supplement: Supplementary Figure 3 — Expression of costimulatory molecules in conventional hmoDCs and WIN-hmoDCs. Mean fluorescence intensity (MFI) of the indicated surface molecules expressed by LPS-activated conventional hmoDCs or WIN-hmoDCs (n=7). Values are shown as mean ± SEM. [file Image_3.tif]

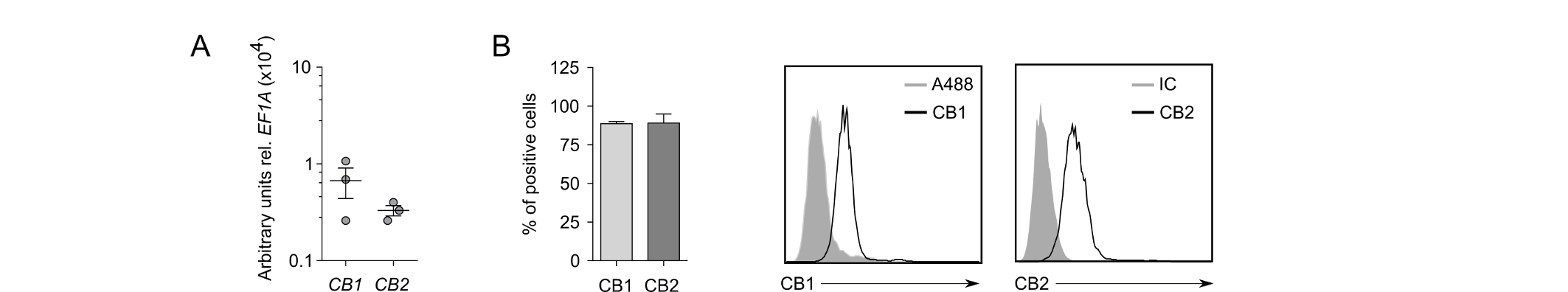

Supplement: Supplementary Figure 4 — Expression of CBRs in human macrophages. mRNA (A) and protein levels (B) of CB1 and CB2 in human macrophages was assayed by qPCR and flow cytometry (n=3). Values are shown as mean ± SEM. [file Image_4.tif]

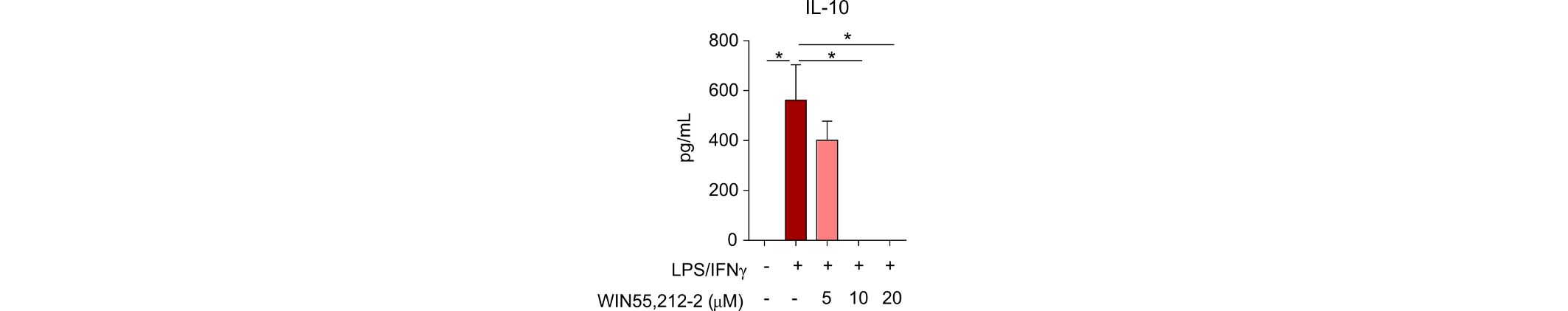

Supplement: Supplementary Figure 5 — WIN55,212-2 effect on IL-10 production. Graph shows IL-10 levels after stimulation of GM-MΦs with medium, LPS/IFNγ (100 ng/mL and 50 ng/mL) or LPS/IFNγ plus the indicated doses of WIN55,212-2 (n=6). Values are shown as mean ± SEM. Statistical significance was determined by One-way ANOVA. * P<0.05. [file Image_5.tif]

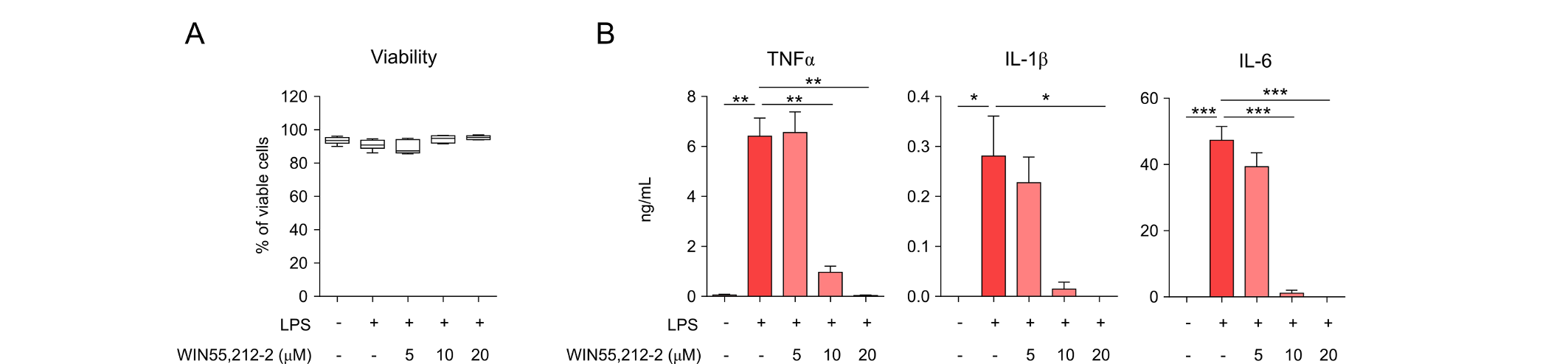

Supplement: Supplementary Figure 6 — Lower doses of LPS induces inflammatory activation of GM-MΦs without affecting cell viability. Percentage of viable cells (A) and cytokine production (B) after stimulation of GM-MΦs with medium, LPS (10 ng/mL) or LPS plus the indicated doses of WIN55,212-2 (n=6). Values are shown as mean ± SEM. Statistical significance was determined by One-way ANOVA. * P<0.05, ** P<0.01 and *** P<0.001. [file Image_6.tif]

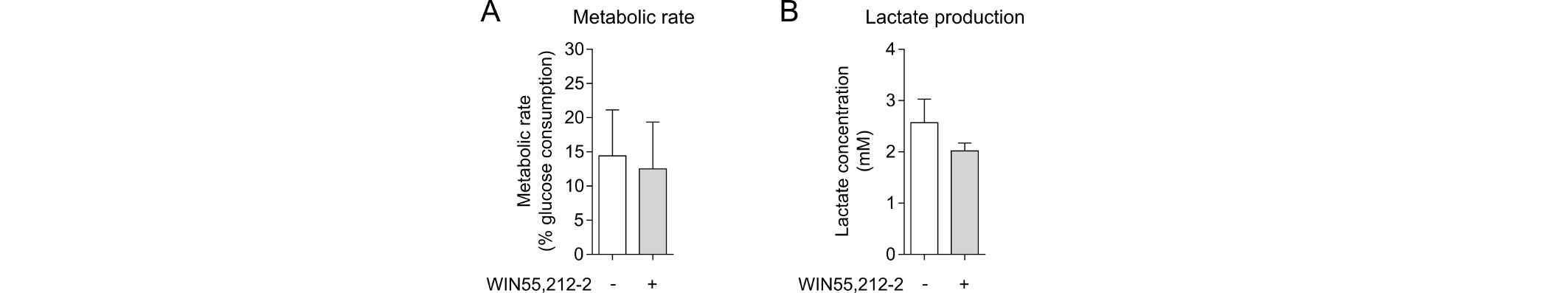

Supplement: Supplementary Figure 7 — Metabolic changes induced by WIN55,212-2 administration in human macrophages. Percentage of glucose consumed (A) and lactate produced (B) by unstimulated or WIN55,212-2 activated GM-MOs (n=2). Values are shown as mean ± SEM. [file Image_7.tif]

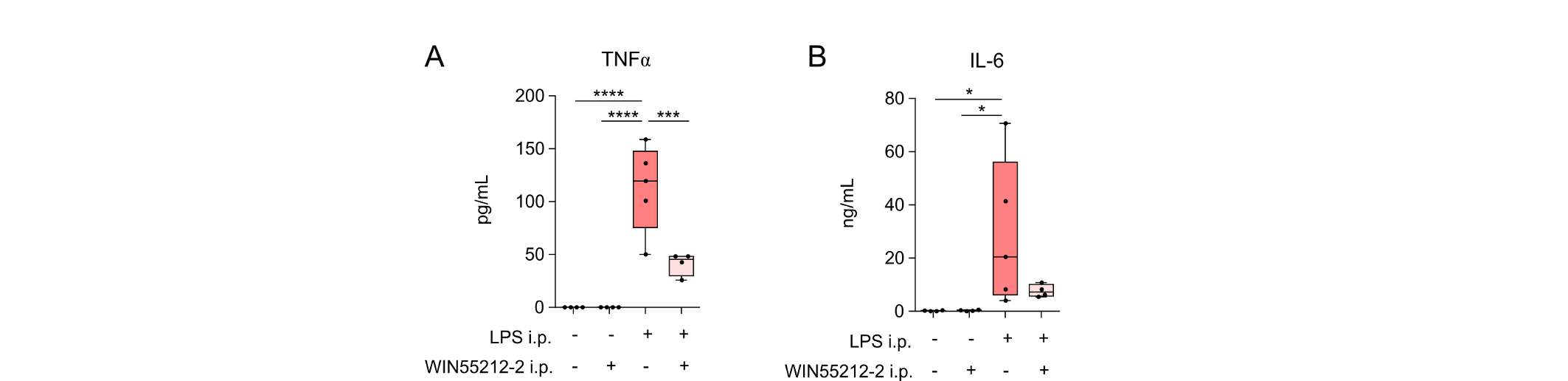

Supplement: Supplementary Figure 8 — WIN55,212-2 inhibits cytokine production in LPS-induced septic mice. Serum levels of TNFα (A) and IL-6 (B) after 12h of intraperitoneal (i.p.) administration of PBS (Control), WIN55,212-2 (5mg/kg), LPS (20mg/kg) or LPS plus WIN55,212-2 (n=4-5). Values are shown as mean ± SEM. Statistical significance was determined by One-way ANOVA. * P<0.05, ** P<0.01, *** P<0.001 and **** P<0.0001. [file Image_8.tif]
