# Supplementary material for: Cannabinoid WIN55,212-2 reprograms monocytes and macrophages to inhibit LPS-induced inflammation
Source: Front Immunol. 2023 Mar 16;14:1147520. doi: 10.3389/fimmu.2023.1147520 (PMC10060516; doi:10.3389/fimmu.2023.1147520)
Supplement: Supplementary file 9 [file Table_1.docx]

| Gene target | Forward primer sequence | Reverse primer sequence |
| --- | --- | --- |
| *CD80* | GATAACCTGCTCCCATCCTGG | CTTGGGGCAAAGCAGTAGGT |
| *CCR7* | CATGGACCTGGGGAAACCAA | AAAGTTCCGCACGTCCTTCT |
| *IL1B* | TTCGACACATGGGATAACGAGG | TTTTTGCTGTGAGTCCCGGAG |
| *IL6* | GGTACATCCTCGACGGCATCT | GTGCCTCTTTGCTGCTTTCAC |
| *IL8* | GCAGCTCTGTGTGAAGGTGCAGTT | TTCTGTGTTGGCGCAGTGTGGTC |
| *NLRP3* | TTGAGAACTGTCATCGGGTGG | CGCCCCAACCCATGAGAAC |
| *CASP1* | AGGATATGGAAACAAAAGTCGGC | AGATAATGAGAGCAAGACGTGTG |
| *GSDMD* | ACAACTTCCTGACAGATGGGG | GGCACAGCTCTCTGTCCAA |
| *HK2* | TTCGCACTGAGTTTGACCAG | TCACCAGGATAAGCCTCACC |
| *PFKB3* | AGCCCGGATTACAAAGACTGC | GGTAGCTGGCTTCATAGCAAC |
| *LDHA* | ATGGCAACTCTAAAGGATCAGC | CCAACCCCAACAACTGTAATCT |
| *HIF1A* | TCGCATCTTGATAAGGCCTCT | ACAAAACCATCCAAGGCTTTCA |
| *EF1A* | CTGAACCATCCAGGCCAAAT | GCCGTGTGCAATCCAAT |
| *TNF* (ChIP) | ACCACGCTCTTCTGCCTGCT | TCCATCCCTCCCTATCAGCGCA |
| *IL1B* (ChIP) | GGCCACCACCACCAACGTTA | AAGGGAGTTCTGGGCCACTTTG |
| *IL6* (ChIP) | ACGCCTTGAAGTAACTGCACGA | TTCCTCTGACTCCATCGCAGCC |

**Supplementary table 1**. List of oligonucleotides used for qPCR.
